# Acidified manure and nitrogen-enriched biochar showed short-term agronomic benefits on cotton–wheat cropping systems under alkaline arid field conditions

**DOI:** 10.1038/s41598-023-48996-4

**Published:** 2023-12-15

**Authors:** Suleman Haider Shah, Muhammad Baqir Hussain, Ghulam Haider, Tanveer Ul Haq, Zahir Ahmad Zahir, Subhan Danish, Bilal Ahamad Paray, Claudia Kammann

**Affiliations:** 1 Department of Soil and Environmental Sciences, Muhammad Nawaz Shareef University of Agriculture, Multan, Pakistan; 2 Department of Plant Biotechnology, Atta-Ur-Rahman School of Applied Biosciences (ASAB), National University of Sciences and Technology (NUST), Islamabad, Pakistan; 3 Institute of Soil and Environmental Sciences, University of Agriculture, Faisalabad, Pakistan; 4 Department of Soil Science, Faculty of Agricultural Sciences and Technology, Bahauddin Zakariya University, Multan, Punjab Pakistan; 5 Department of Zoology, College of Science, King Saud University, PO Box 2455, Riyadh, 11451 Saudi Arabia; 6 Climate Change Research for Special Crops, Department of Applied Ecology, Hochschule Geisenheim University, Von-Lade Str. 1, 65366 Geisenheim, Germany

**Keywords:** Plant sciences, Plant stress responses, Salt

## Abstract

Application of organic residues such as farm manure and biochar in various agricultural environments have shown positive effects on soil carbon sequestration. However, there is a lack of consensus regarding the agronomical benefits of a single and small dose of biochar and farm manure in arid alkaline soils. Therefore, a field experiment with the given treatments (1) control (no amendment), (2) acidified manure (AM) at 300 kg ha^−1^, (3) nitrogen (N) enriched biochar (NeB) at 3 Mg ha^−1^, and (4) an equal combination of AM + NeB (150 kg ha^−1^ AM + 1.5 Mg ha^−1^ NeB)) was conducted in a typical cotton–wheat cropping system. A parallel laboratory incubation study with the same amendments was carried out to account for soil carbon dioxide emission (CO_2_). The N enrichment of biochar and its co-application with acidified manure increased soil mineral N (NO_3_^−^ and NH_4_^+^) in the topsoil (0–15 cm), and increased total N uptake (25.92% to 69.91%) in cotton over control, thus reducing N losses and increased uptake over control. Compared to the control, co-application of AM + NeB significantly improved soil N and P bioavailability, leading to increased plant biomass N, P, and K (32%, 40%, 6%, respectively) uptake over control. The plant's physiological and growth improvements [chlorophyll (+ 28.2%), height (+ 47%), leaf area (+ 17%), number of bolls (+ 7%), and average boll weight (+ 8%)] increased the agronomic yield in the first-season crop cotton by 25%. However, no positive response was observed in the second season wheat crop. This field study improved our understanding that co-application of acidified manure and N-enriched biochar in small dose can be a strategy to achieve short-term agronomic benefits and carbon sequestration in the long run.

## Introduction

Soil organic carbon plays an integral role in the processes involved in the maintenance of soil health and fertility, but it is on a declining trajectory in arid alkaline soils. Biochar is such a method for increasing and sequestering soil carbon, as several primary research and meta-analysis studies have found that biochar addition can trigger additional SOC build-up beyond the initial biochar addition^1–3^ and reduce greenhouse gas emissions^4,5^. Furthermore, several recent review/meta-analysis studies have reported that biochar amendments can improve soil physical, chemical, and biological properties^6–10^ improve soil nutrient transformation^11,12^, agronomic yield especially in degraded/poorly fertile soils of tropics^13,14^ but may also decrease/not improve crop yields due to nutrient uptake limitations^15^. Jeffery et al.^14^ reported greater chances of an increase in crop productivity by biochar application in the tropics compared to fertile soils in temperate regions. Recently, in another meta-analysis, Ye et al.^16^ reported a 31% increase in crop yield by the application of biochar in marginal soils in conjunction with inorganic fertilizers in short-term field studies.

However, it has been widely investigated that the use of high amounts (> 10 Mg ha^−1^) of biochar in soil is neither agronomically nor financially viable^17–21^. In the United States, the cost of biochar for agricultural use ranges from $300 to $500 Mg^−1^^22^, while in Europe, the cost is €200 to €600 Mg^−1^^23^. Furthermore, small farm holders have limited access to biochar, with only one Mg of biochar available per household per year due to low recovery and unavailability of feedstock^24,25^. As a result, recent trends in biochar application have shifted away from large doses to low doses through biochar value addition via impregnation with organic manures, composts, or synthetic fertilizers. A study reported a 5 to 15% increase in crop yield from a field study with a biochar application rate of 1 to 2 Mg ha^−1^^26^. In a pot trial, a low dose of biochar (0.5 Mg ha^−1^) mixed with NPK fertilizers increased wheat seedling biomass by 12 to 20% over NPK application alone^18^. Furthermore, Kong et al.^27^ found that applying biochar at 3 Mg ha^−1^ increased seed cotton yield by 8.4 to 22.8%, and P availability by 5.5 to 12.1%. Pandit et al.^28^ demonstrated average yield increases of 100% in 21 field experiments in Nepal by root zone application of organically or mineral-fertilizer enriched biochar amounts of 0.7–2 Mg ha^−1^.

Co-application of biochar with farmyard manure and poultry manure as phosphorous sources in combination with inorganic phosphorous or as N sources significantly improved wheat and cotton yield under different climatic conditions of Pakistan^29,30^. Thus, returning organic residues (straw, compost, manure, biochar) into the field by engineering the biochar properties for specialized objectives under varying soil and environmental conditions has the potential to develop environmentally friendly agronomic strategies to improve soil carbon sequestration, crop yields, and to reduce agricultural greenhouse gases emissions.

In this study, field and laboratory incubation experiments were established to investigate the impacts of low doses of acidified manure and nitrogen-enriched biochar on poorly fertile alkaline sandy loam soil in a dry, semi-arid region of Pakistan. The two growing seasons (2018–2019) field experiment was conducted in a cotton (*Gossypium hirsutum*) wheat (*Triticum aestivum*) rotation system in South Punjab. We aimed to find out whether (1) manure acidification, (2) nitrogen enrichment of biochar, and (3) low doses application in the root zone instead of broadcasting have short-term agronomic effects on crop production, and if effects will persist in the follow-up crop, to justify treated biochar recommendations to the farmers and other stakeholders’ interest in the wider use of biochar for improving soil organic matter, crop production and environmental benefits. The effects of treated biochar and manure were investigated on crop growth, physiology, nutrient uptake, yield, nitrate leaching under field, and soil CO_2_ emission under incubation conditions.

## Materials and methods

### Study site

**Figure 1:**
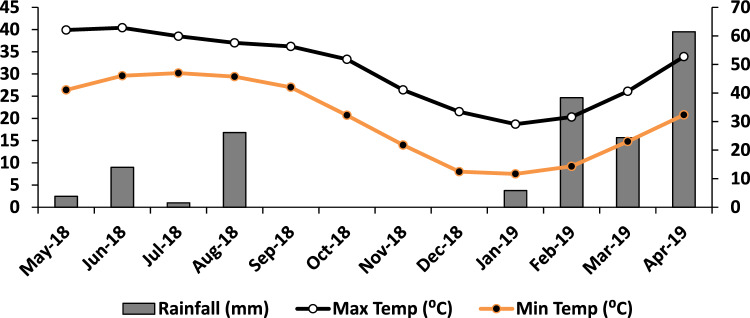
Monthly average minimum and maximum temperature and rainfall during the cotton-wheat cropping years.

The experimental farm of the Muhammad Nawaz Sharif University of Agriculture Multan is located at 71° 26′ 39.21″ E and 30° 8′ 24.43″ N. The study site falls in cotton mix cropping zone—VII of the province Punjab, Pakistan. The region’s agricultural land is mainly occupied by the major crops cotton and wheat, but also mango orchards, vegetables, and fodders for livestock development. Temperature and rainfall data during study duration is presented in Fig. 1.

### Characterization of soil, biochar, and acidified manure

A composite soil sample was taken from the experimental field at (0–30 cm) depth after the harvest of the previous wheat crop and before seedbed preparation for the next cotton crop following the method explained in the Soil and Plant Analysis Laboratory Manual^31^. The air dried and sieved (2 mm) soil samples were analyzed for soil physico-chemical properties following the procedures described in the Soil and Plant Analysis Laboratory Manual^31^ and presented in Table 1.

**Table 1:** Physicochemical traits of soil and biochar.

Physicochemical Traits	Unit	Soil	Biochar (DWB)
Textural class	–	Sandy clay loam	–
Saturation percentage	%	35	–
EC	dS m^−1^	0.73	1.47
pH	–	8.5	8.5
Organic matter	%	0.34	–
Total Nitrogen	%	0.01	0.93
Phosphorus	–	2.1 mg kg^−1^ (Olsen-P)	0.20%
Potassium	–	112 mg kg^−1^ (Plant available)	1%
Ash content	% (w/w)	–	9.1
Carbon	% (w/w)	–	88.6

DWB, dry weight basis; EC, electrical conductivity.

The pruning and wood bark waste of *Acacia arabica* (local name Kikar) were collected from the wood industry and dried in the open via irradiation for 7 days. Biochar was produced by using a novel Kon-Tiki Flame Curtin Pyrolysis Kiln^32^. Briefly, a small chimney of dried woods was established and burned in the bottom of the kiln. As the smokeless flames started to decline (indicating that wood gas outgassing was nearly complete), new layers of feedstock were applied on top of the glowing ember charcoal bed which led to renewed outgassing and a flame curtain that prevented the biochar forming below from turning into ash. When the cone was nearly filled, the pyrolysis reaction was quenched by applying water from the top. The temperature of the main pyrolysis zone is 680° to 750 °C and when new feedstock is added it cool down and temperature goes down to 150–450 °C. The pristine biochar was characterized for detailed chemical properties by Eurofins Umwelt Ost GmbH (Bobritzsch-Hilbersdorf), Germany; results are reported in Table 1. (For the procedure of N enrichment of the biochar see 2.3.) Acidified manure (pH 3.5) was prepared at the Soil Microbiology and Biochemistry Laboratory, Institute of Soil and Environmental Sciences, University of Agriculture, Faisalabad, Pakistan and its manufacturing procedure and composition is described by Abbas et al.^33^. Briefly, mixed manure from cows and goats were obtained from a university agricultural farm. The manure contains carbon (55.4%), nitrogen (1.87%), and phosphorus (0.34%). The manure was acidified by the bio-augmentation process using Sulphur oxidizing bacteria along with elemental sulfur and incubated for 25–30 days as adopted by Abbas et al.^33^, and the final pH of the acidified manure was pH = 3.5.

### Field experimental setup

The experiment was conducted consecutively for two cropping seasons by sowing cotton in spring 2018 and wheat in fall 2018–2019. The experimental field was prepared by plowing, followed by leveling and planking. Seedbeds were prepared with a specialized tractor-mounted cotton bed-planter. The experimental plots demarcation (each measuring 5 × 3 m^2^, length × width) was carried out after bed preparation. The experimental treatments included (1) control, no biochar or manure application, (2) acidified manure at 300 kg ha^−1^ (AM), (3) nitrogen-enriched biochar at 3 Mg ha^−1^ (NeB), (4) acidified manure at 300 kg ha^−1^ + nitrogen-enriched biochar at 3 Mg ha^−1^ (AM + NeB). Biochar and acidified manure were applied on a dry weight basis. The nitrogen (N), phosphorus (P), and potassium (K) fertilizers were applied at rates of 170, 60, and 50 kg ha^−1^ (N, P, and K, respectively). For manure treatment plot, the N, P, and K was applied after deducting the amounts of nutrients in manure. For the NeB treatment, the basal dose of N as urea (1/3rd of 170 kg) was dissolved in water, and biochar was soaked in this solution in such a way that it absorbed the solution completely. For the treatment AM + NeB, nitrogen-enriched biochar was mixed with acidified manure before soil application. Following N enrichment, biochar or biochar + manure was immediately incorporated in root-zone trenches at the cotton seedbeds. The trenches on the beds were immediately closed after putting treatment materials in respective plots. The basal dose of N fertilizer for the control and acidified manure treatments was applied along with the complete doses of P and K fertilizers to all remaining plots. The experimental plots were laid out according to randomized complete block design (RCBD) with three replications. Cotton seeds of a Bt variety IUB-2013 were planted on 28 May 2018 manually on beds with a planting geometry of 30 cm plant to plant and 75 cm row to row spacing. Weeds were controlled by the soil application of pre-emergence herbicide ‘Pendimethalin 33% EC’ after 16 h of seed sowing. Initial irrigation was applied on the day of seed sowing on beds, the subsequent irrigations were applied at 7 to 12 days intervals depending on the soil moisture and weather conditions. The cotton was harvested twice in October and in November. A treatment-wise soil sampling was performed to check the available mineral elements. No biochar or manure was applied in the second season, however complete doses of N (150 kg ha^−1^) as urea, P (100 kg ha^−1^) as diammonium phosphate, and K (60 kg ha^−1^) as sulphate of potash were applied in the respective experimental plots. The N was applied in two splits à 75 kg ha^−1^ at sowing and just before the first irrigation at day 21 after sowing. The wheat, variety Galaxy-2013, was planted with a tractor-mounted drill machine at 30 cm row to row spacing at a seed rate of 124 kg per hectare on 05 December 2018.

### Crop husbandry

Cotton growth and development measurements were performed on plants in the central part of the experimental plot to avoid border effects. Leaf chlorophyll contents (SPAD values) were measured at 90 days after sowing (DAS) on the 4th fully developed leaf using a SPAD-502 (Konica-Minolta, Japan). Plant height, codes per plant, bolls per plant and height to node ratio were counted and calculated. Leaf area was measured following the procedure described by Monteiro et al.^34^ using a leaf area meter CI-202 (CID Bio-Science, Inc., USA) in the laboratory. Leaf area index (LAI) was calculated using the formula: LAI = (leaf area per plant x number of plants) / plant area (2 m^2^). Seed cotton was harvested twice (during October-2018 and November-2018.

For wheat, the chlorophyll content (SPAD value) and LAI were measured at 90 DAS. Chlorophyll content was recorded with three repeated readings per flag leaf using a SPAD-502 device (Konica-Minolta, Japan). Leaf area was measured following the procedure of Yin et al.^35^ and using a leaf area meter (CI-202, CID Bio-Science, Inc., USA). The data of yield and yield contributing parameters plant height, spike length, grains per spike, spikelets per spike, plant biomass, and grain yield were recorded was recorded at maturity and following standard procedures.

### Soil analysis for NH_4_–N, NO_3_–N, and Olsen P

For the determination of mineral N (N–NH_4_^+^ and N–NO_3_^−^) concentrations in soil at different depths (0–15 cm, 15–30 cm, and 30–60 cm), soil samples were collected at the time of harvest for each crop (cotton and wheat, respectively). The fresh soil samples were packed in plastic bags, stored in cooling boxes, and immediately transferred to the laboratory for mineral N extraction. The mineral N (N–NH_4_^+^ and N–NO_3_^−^) was extracted from 10 g of soil with 2 M KCL solution and the extract was stored at 4°C^36^. For the determination of N–NO_3_^−^ in soil extract, the salicylic method as described by Cataldo et al.^37^ was followed. For the determination of N–NH_4_^+^, the procedure described by Keeney et al.^38^ was followed.

Soil saved from the soil samples taken at 0–15 and 15–30 cm depth of each treatment plot was pooled to have a homogeneous mixture representing a soil depth of 0–30 cm and was used for measurement of plant-available P (Olsen P) by using the method of Watanabe and Olsen^39^.

### Plant nutrient analysis

Cotton and wheat plant samples (excluding seed cotton and grain, respectively) were collected from each plot at maturity. Plant samples were oven-dried at 65 ± 1 °C and ground to 2 mm. The ground samples (0.5 g) were digested with concentrated sulfuric acid and hydrogen peroxide (Wolf, 1982). The digested samples were analyzed for nitrogen by the Kjeldahl method, phosphorus by using a spectrophotometer (CE 7400S, Cecil Aquarius, Cecil Instruments Limited, Cambridge, UK) at wavelength 410 nm^40^, and potassium by a flame photometer (Jenway PFP-7, England) using calibration curve^31^.

### Incubation experiment for the estimation of CO_2_ emission

A parallel incubation experiment was conducted to estimate the CO_2_ emissions from the soil after amendment with the same treatments that were used in the field experiment. The applied method for measuring soil respiration was followed as described by Isermeyer et al.^41^. Briefly, the sieved field soil with 60% water holding capacity was weighed (50 g) and treated with the amendments (equivalent to the field treatment plan), and placed in 200 ml jars. The soil treatments were arranged in a completely randomized design (CRD) with 4 replications (*4* × *4, n* = *16*) and pre-incubated at 25 ± 1 °C for 7 days. After every three days, the soil moisture was corrected on a soil moisture loss basis from a target weight. After incubation, each treatment jar was placed at the bottom of a 1L (L) plastic jar containing 25 ml of 0.05 molar (M) NaOH. The lids of the 1L jars were closed immediately to make it airtight and incubated at 25 ± 1 °C. The jars without soil served as a blank. After 3 days of incubation, jars were opened and NaOH was titrated against 0.05 M HCl after adding 5 ml of 0.5 M BaCl_2_ and a few drops of an indicator (phenolphthalein), till the color changed from red to colorless. The same process was repeated with fresh NaOH solution after every 3 days for a total incubation time of 60 days. The following equation was used to calculate the rate of CO_2_ evolved from the treatment soils.

$$ \begin{aligned} & {\text{CO}}_{{2}} \;\left( {{\text{mg}}} \right)\;{\text{evolved}} = \left( {{\text{V}}_{0} {-}{\text{V}}_{{1}} } \right) \times {1}.{1} \\ & \quad \quad \quad \quad \quad \quad \quad \quad \quad {\text{D}}_{{{\text{wt}}}} \\ \end{aligned} $$

where V_0_ = HCl used for blank, V_1_ = HCl used for treated soil sample, D_wt_ = dry weight of 1 g moist soil, 1.1 = is the conversion factor as 1 ml of 0.05 M NaOH is equal to 1.1 mg CO_2_.

### Statistical analysis

Analysis of variance (ANOVA) was applied for statistical analysis of the data (Steel, R., 1997), and treatment means were compared using the Least Significant Difference (LSD) test at *p* ≤ 0.05 (*n* = *3 for the field trial and n* = *4 for the incubation experiment*) to identify the significant differences among treatment means. Randomized Complete Block Design (RCBD) was used for the field study and Completely Randomized Design (CRD) was used for the incubation experiment. The statistical analyses were performed by using the software Statistics 8.1 (Analytical Software, Informer Technologies, Inc.).

### Ethics approval and consent to participate

We all declare that manuscript reporting studies do not involve any human participants, human data, or human tissue. No approval/ permissions/licenses is required for cotton plants because plants were collected from university research area. So, it is not applicable.

### Study protocol must comply with relevant institutional, national, and international guidelines and legislation

The use of plants in the present study complies with international, national and/or institutional guidelines. 

## Results

### Crop growth, physiology, and agronomic yield

**Figure 2:**
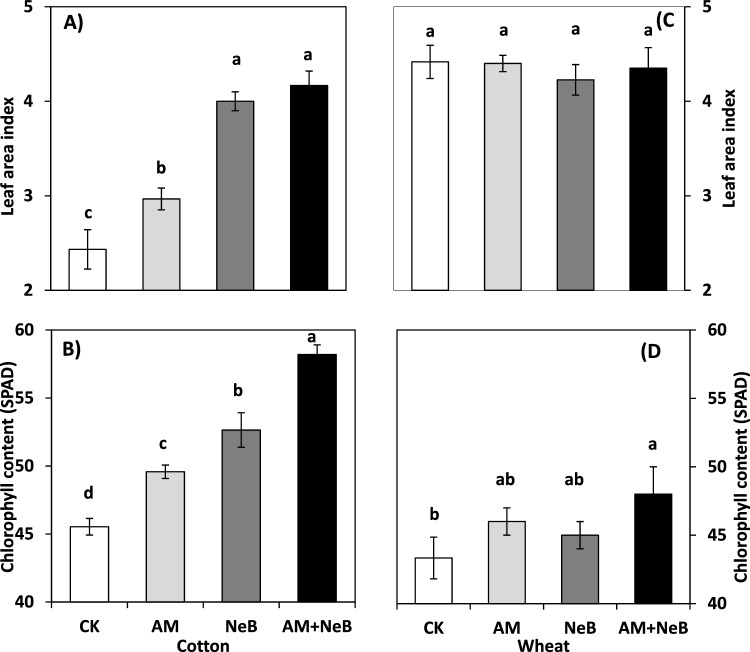
Effect of different treatments on A = leaf area index of cotton at 120 DAS, B = chlorophyll content of cotton at 90 DAS, C = leaf area index of wheat at 120 DAS and D = wheat chlorophyll content of wheat at 90 DAS. Here DAS = days after sowing, CK = control (no AM or NeB), AM = acidified manure, NeB = nitrogen enriched biochar and AM + NeB = combination of acidified manure and nitrogen enriched biochar. The vertical bars indicate treatment means, while error bars show the standard deviation of means (SD; n = 3). The bars sharing similar letters are not significantly different from each other at *p* ≤ 0.05.

The application of nitrogen-enriched biochar ‘NeB’ and acidified manure ‘AM’ or their combination improved cotton growth, physiology, and yield contributing traits (Figs. 2A, 4B; Table 2). However, the combined application of AM and NeB surpassed the effect of their sole application and improved plant growth, physiology, and yield significantly. For instance, the combined application increased leaf chlorophyll content by 28.2%, plant height by 47%, leaf area index 17% and 13% (at 90 and 120 days after sowing, respectively), the number of bolls per plant by 7%, and average boll weight by 8% over that of the control treatment with no manure or biochar amendment. The growth and physiological improvements resulted in enhanced agronomic yield (seed cotton yield) of 25% over that of the control.

**Table 2:** Effect of treatments on cotton yield and yield contributing parameters.

Treatments	Plant height (cm)	Nodes per plant (No.)	Height to node ratio	Bolls per plant (No.)	Average boll weight (g)	Seed cotton yield (Mg ha^−1^)
CK	82 ± 7c	33.7 ± 1.2a	2.52 ± 0.22c	35.67 ± 2.31c	3.15 ± 0.07b	2.01 ± 0.19c
AM	88 ± 2c	32.0 ± 2.0a	2.77 ± 0.13bc	38.83 ± 0.55b	3.30 ± 0.03ab	2.31 ± 0.10ab
NeB	110 ± 1b	38.0 ± 2.0a	2.89 ± 0.13b	40.10 ± 0.66b	3.17 ± 0.08b	2.20 ± 0.13bc
AM + NeB	121 ± 3a	35.0 ± 1.0a	3.46 ± 0.17a	42.57 ± 0.67a	3.40 ± 0.12a	2.51 ± 0.12a
*P* values	0.0000	0.0183	0.0041	0.0025	0.0095	0.0476

	Plant height (cm)	Spike length (cm)	Grains per spike	Spikelet per spike	Biomass yield (Mg/ha)	Grain yield (Mg/ha)
CK	95.00 ± 0.00a	11.33 ± 0.58a	37.33 ± 3.06c	14.17 ± 1.04b	7.68 ± 0.23c	3.86 ± 0.42a
AM	88.33 ± 5.03a	10.33 ± 0.58a	47.00 ± 2.00b	17.83 ± 1.04a	9.08 ± 0.21a	4.66 ± 1.36a
NeB	92.67 ± 1.53a	11.00 ± 1.00a	39.00 ± 1.00c	14.83 ± 0.29b	8.24 ± 0.16bc	3.76 ± 0.34a
AM + NeB	90.00 ± 5.20a	10.00 ± 1.00a	54.67 ± 4.16a	17.83 ± 0.29a	8.84 ± 0.70ab	5.37 ± 0.48a
*P* values	0.2143	0.3241	0.0018	0.0013	0.0217	0.1699

Values in the columns are means ± standard deviation (n = 3). Here CK = control (no AM or NeB), AM = acidified manure, NeB = nitrogen enriched biochar and AM + NeB = combination of acidified manure and nitrogen enriched biochar. The values presented in the table are treatment means. The treatment means sharing similar letters are not significantly different from each other at *P* < 0.05.

The residual effects of the treatments on wheat growth and physiological traits were inconsistent (*p* ≤ 0.05; Table 2, Fig. 2C,D). The sole application of AM or its co-application with NeB showed significant increase in chlorophyll content, spikelet per spike, grains per spike, and total biomass yield. However, the magnitude of these improvements was not enough to convert it into a significantly higher grain yield over that of the control Fig. 2C.

### Plant biomass element (N, P, and K) uptake at harvest

**Figure 3:**
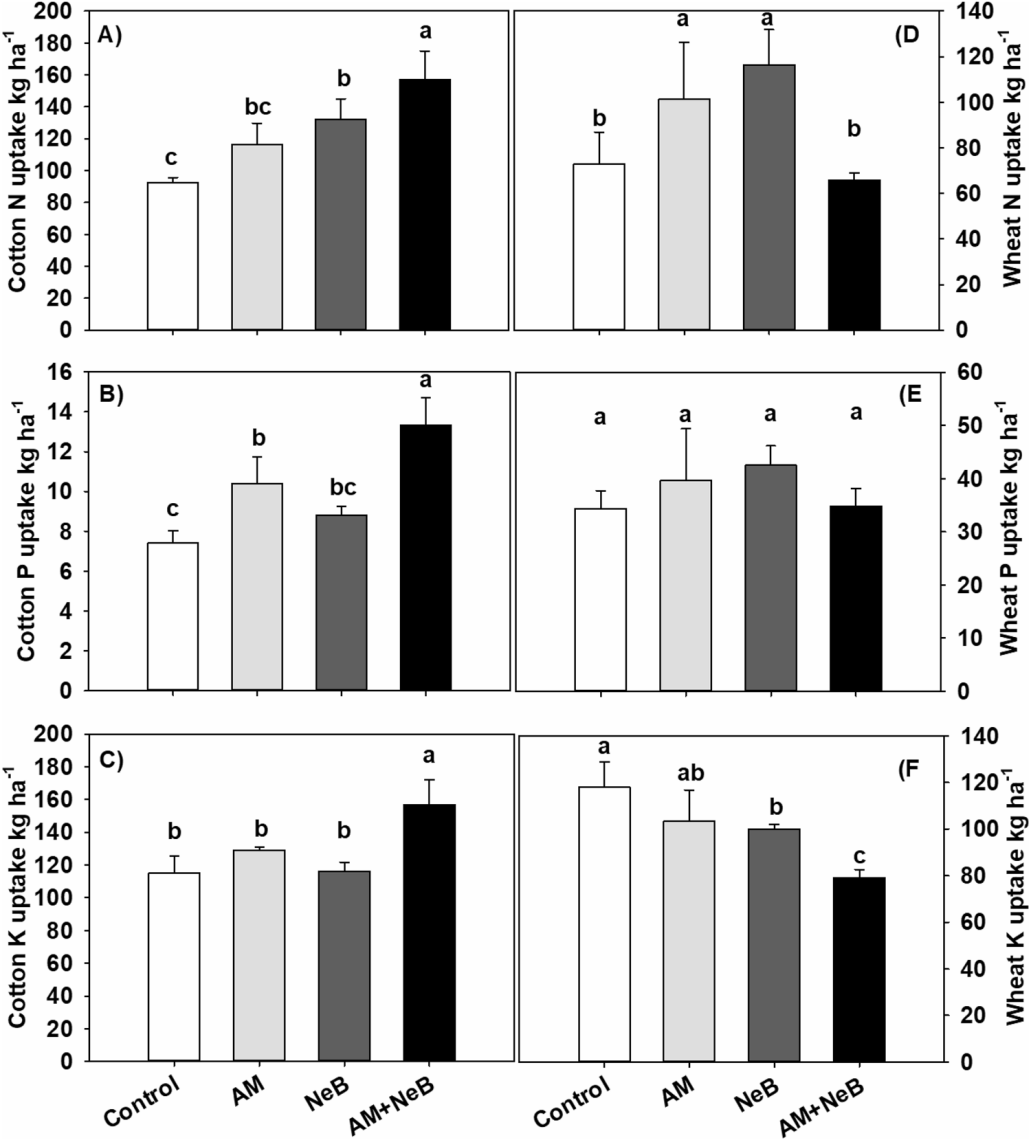
Effect of different organic treatments cotton and wheat nutrient uptake in consecutive cropping seasons. Control = no organic amendment, AM = acidified manure, NeB = nitrogen enriched biochar and AM + NeB = combination of acidified manure and nitrogen enriched biochar (50:50 w/w basis of only AM or NeB). The bar in the figure indicates the treatment means, while error bars show the standard deviation of means. The bars sharing similar letters are not significantly different from each other at *p* ≤ 0.05.

The organic treatments significantly affected cotton plant biomass (excluding seed cotton) total N, P, and K uptake compared to the control (Fig. 3). However, the combined application of AM and NeB during the first cropping season showed the highest increase in cotton plant biomass N, P, and K uptake (+ 69.9%, + 79.6%, and + 36.5%, respectively) compared to sole AM, NeB, or control treatment. However, during the second growing season, (where only the recommended fertilizer doses for the crop (wheat) were applied without organic amendments (AM or NeB)), there were no consistent effects regarding wheat nutrient uptake (Fig. 3). Wheat total N uptake was increased by the sole application of AM (+ 39.1%) and NeB (+ 59.8%), however, their combined (AM + NeB) application reduced uptake of N (by − 9.5%) over that of the control treatment. There was no significant improvement in wheat P uptake by the application of all organic amendments. However, the wheat total K uptake was significantly decreased by − 2.3%, − 7.9%, and − 31.4% over control due to AM, NeB, or their combined application as AM + NeB, respectively.

### Soil mineral nitrogen “NO_3_^−^–N and NH_4_^+^–N” (N_min_)

**Figure 4:**
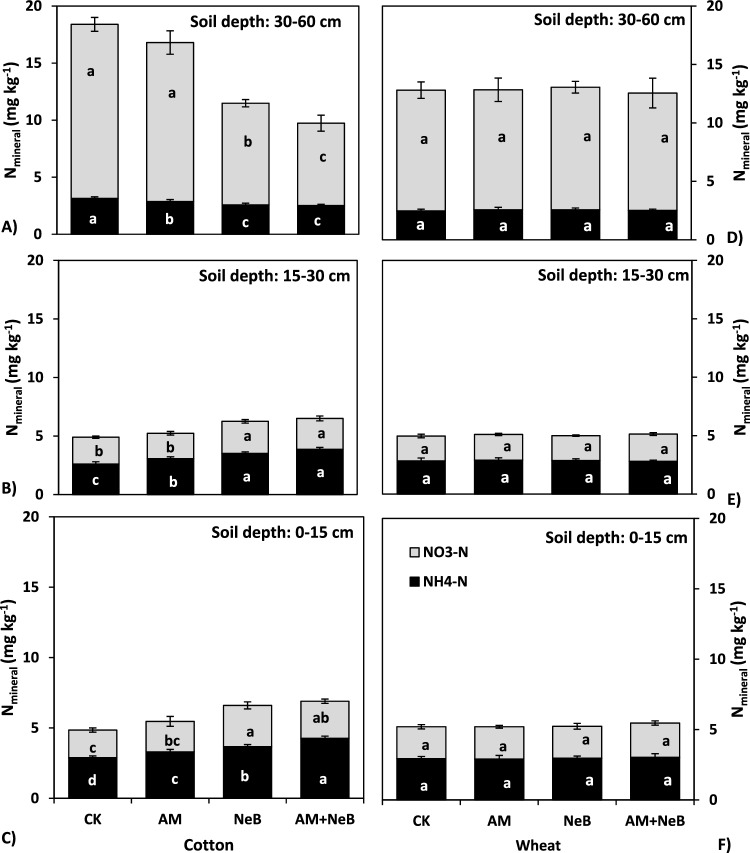
Effect of different treatments on soil mineral nitrogen (NH_4_^+^-N and NO_3_^—^N) at different soil depths (0–15 cm, 15–30 cm and 30–60 cm), A = soil mineral N measured at depth 0–15 cm in cotton, B = soil mineral N measured at depth 15–30 cm in cotton, C = soil mineral N measured at depth 30–60 cm in cotton, D = soil mineral N measured at depth 0–15 cm in wheat, E = soil mineral N measured at depth 15–30 cm in wheat and F = soil mineral N measured at depth 30–60 cm in wheat. Here CK = control (no AM or NeB), AM = acidified manure, NeB = nitrogen enriched biochar and AM + NeB = combination of acidified manure and nitrogen enriched biochar. The vertical bars in the figure indicate the treatment means, while error bars show the standard deviation of means (SD; n = 3). The bars sharing similar letters are not significantly different from each other at *p* ≤ 0.05.

Soil N_min_ (NO_3_^−^–N and NH_4_^+^–N) concentrations in treated plots at different soil depths (0–15, 15–30, and 30–90 cm) in the first (summer-fall 2018) and the second (winter-spring 2018–19) crop seasons are presented in Fig. 4. During the first crop season, all the treatments increased (*p* ≤ 0.05) soil NH_4_^+^-N concentration by 14 to 48% at 0–15 cm soil depth over the concentrations in the control soil (Fig. 4A). The increases followed the order of AM + NeB > NeB > AM > control. A similar trend of NH_4_^+^-N concentration was observed at 15–30 cm soil depth (Fig. 4B). However, here, soil NH_4_^+^-N concentration was decreased by all three treatments over that of the control soil layer at 30–60 cm soil depth. Application of NeB and its combination with AM significantly increased soil nitrate concentration at 0–15 cm (34–49%) and also in 15–30 cm (14–19%) soil depth, respectively, however, the same treatments showed significantly lower (42–53%) nitrate concentrations at 30–60 cm depth (Fig. 4A–C). However, there was no lingering residual effect of the treatments compared to the control on soil mineral N concentrations in the succeeding crop season when wheat was grown (Fig. 4D–F).

### Soil P availability

**Figure 5:**
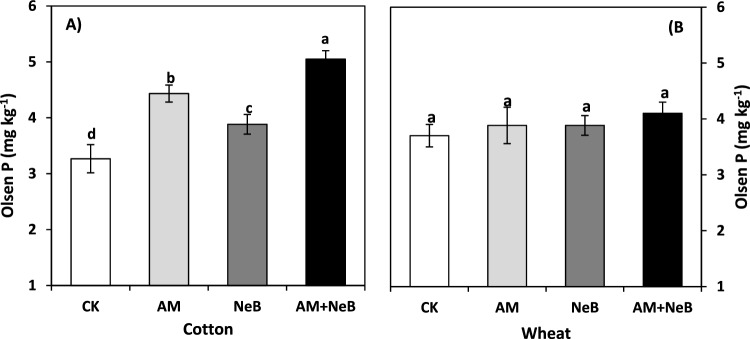
A = Olsen P in cotton and B = Olsen P in wheat. Here CK = control (no AM or NeB), AM = acidified manure, NeB = nitrogen enriched biochar and AM + NeB = combination of acidified manure and nitrogen enriched biochar. The vertical bars indicate treatment means, while error bars show the standard deviation of means (SD; n = 3). The bars sharing similar letters are not significantly different from each other at *p* ≤ 0.05.

All treatments increased soil P availability (Olsen’s P, *p* ≤ 0.05) in the order of NeB + AM (54.6%) > AM (35.7%) and > NeB (18.9%) as compared to the control treatment during the first growing season after the initial treatment applications (Fig. 5A). However, no visible residual effects of the treatments remained in the second crop (wheat) without another treatment application (Fig. 5B).

### CO_2_ emission from soil

**Figure 6:**
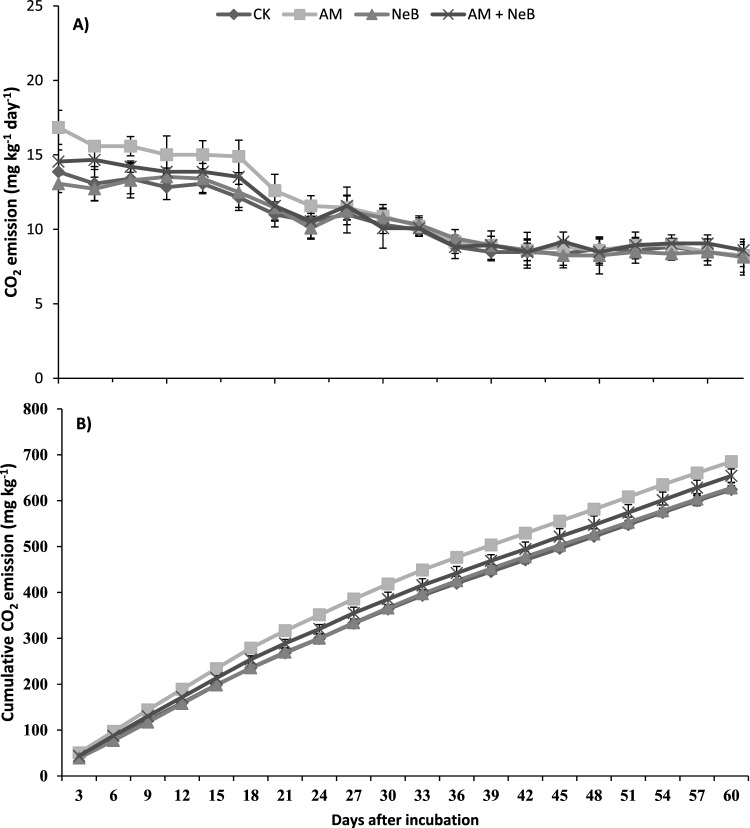
Effect of different treatments on A = CO_2_ emission rate and B = cumulative CO_2_ emission from soil measured at different incubation days after treatment application. Here CK = control (no AM or NeB), AM = acidified manure, NeB = nitrogen enriched biochar and AM + NeB = combination of acidified manure and nitrogen enriched biochar. Error bars represent the standard deviation of means (SD; n = 4).

In all treatments, the CO_2_ emission rate was higher during the first 18 days of incubation and then started to decline till the 36th day of incubation Fig. 6A. The lowest rate of CO_2_ emission starting from the 36th day of incubation remains consistent till the end of the study harvested at the 60th day of incubation. The soil amended with AM exhibited the highest CO_2_ emission rate during the initial 36 days. However, there was no significant difference in the CO_2_ emission rate among all treatments at the end of the incubation period. The cumulative CO_2_ emission sum was higher in soil amended with AM alone, and it was followed by the co-application of AM + NeB. The effect of NeB alone on the CO_2_ emission rate and cumulative CO_2_ emission sum was similar to that of the control. However, when AM was combined with NeB, the CO_2_ emission was reduced as compared to AM sole application.

## Discussion

In this study, low doses of acidified manure (AM) and nitrogen-enriched biochar (NeB) were used to explore their main and residual effects on the cotton–wheat cropping system for two consecutive seasons (2018–2019) in south Punjab, Pakistan.

### Growth and yield response of cotton–wheat following organic amendments

Crop yield as influenced by the application of AM and NeB was increased only during the first season of treatment application in this study. Concerning geographical location, the yield improvement results are in agreement with meta-analysis studies by Jeffery et al.^14^ and Ye et al.^16^ which concluded that yield increases due to biochar amendments are more pertinent in less fertile soils of the tropics. Furthermore, the greater yield due to AM + NeB treatment in this study is in line with Schulz et al.^42^ who found greater crop yield by the application of biochar with organic fertilizer compared to biochar application with mineral fertilizer in an infertile tropical sandy soil. However, it has been found in a recent meta-analysis study^43^ that plant productivity response following biochar amendment can be weakened or strengthened due to the combined effect of soil conditions and properties of biochar and organic amendments (pH, cation exchange capacity (CEC), N, carbon–nitrogen ratio (C/N), soil texture, bulk density, etc.). Thus, it explains that the yield improvement effects of biochar can only be achieved when it is already defined that biochar/organic amendment is being applied to ameliorate a certain constraint to crop growth and productivity^44^. In a previous study^45^, found no maize yield improvement in the first and second crop season by the combined application of fresh biochar and farm manure. However, the AM + NeB application in the present study improved nutrient availability and led to increased seed cotton yield by 25% over control. Similar results of improved cotton productivity were obtained in a two-year field study under arid alkaline soils by the combined application of biochar, poultry manure, and farmyard manure in different combinations^46^. However, there was no visible residual effect on crop productivity in the second season in this study. Such types of short-term positive effects, as observed in the present study, were often attributed to the liming effect of alkaline biochar in acidic soils^47,48^ or vice versa^49^, which may eventually vanish over time^50^. Higher rates of biochar application caused yield reduction in the maize-wheat system due to nutritional (e.g. N) deficiency^51^ or showed no positive residual effect on crop yield even after 4 years of application in a temperate climate^52^. Thus, it is the matter of specialized soil, environmental, biochar, or any other organic amendment properties which determine the overall short or longer-term impact on soil health and crop productivity under specialized conditions.

### Elemental composition of crop plants as influenced by acidified manure and nitrogen-enriched biochar

Plant biomass P and N uptake were significantly increased as influenced by the combined application of AM and NeB in the first season (cotton crop) in this study. However, there was no residual positive effect of AM or NeB combinations on wheat nutrient uptake (in the second season) except P, which was significantly increased due to the combined application of AM and NeB. Studies have suggested that composting of biochar or mixing it with organic manures can help to fix biochar’s inherent nutrient deficiency and may improve its chemical properties to ensure required agronomic benefits^46,53–55^. However, it is important to understand that the soil's physical and chemical properties are the regulators of plant nutrient availability. For instance, P availability is a challenge in both alkaline^49^ and acidic^56^ soils due to reaction with Ca^2+^, K^+^, Mg^2+^, and Na^+^ in alkaline and with Al^3+^ or Fe^3+^ in acidic soils. Nitrogen is another limiting nutrient which is also affected by soil properties. Alkaline soils in dry humid regions lead to NH_3_ volatilization^57^ and poor structure, while well-drained coarse soils lead to nitrate leaching^58^. Biochar amendment in agricultural soils has been widely advocated for improving nutrient use efficiency^19^, specifically for nitrogen and phosphorous^59,60^. However, there is no single biochar fit for all conditions^61^ because the responses are governed by properties of biochar defined by pyrolysis temperature^62^ or by the engineering of biochar properties^63^. In the present study, we were partially successful to improve soil conditions by acidification of farm manure and N enrichment of biochar as indicated by greater N and P uptake in the first but with fading effects in the second season and by increased soil mineral N concentrations in the upper soil horizon.

### Effects of acidified manure and nitrogen-enriched biochar on soil mineral nitrogen dynamics

Nitrogen is one of the limiting nutrients regulating plant growth and critical of overall primary productivity in terrestrial ecosystems. However, denitrification and ammonia volatilization loss are major factors associated with N fertilizer use in arid and semi-arid regions with alkaline soils^64^. Therefore, N stabilizers, nitrification inhibitors, and other management strategies are recommended for reducing N losses in agroecosystems^65^. It has been widely reported that biochar amendment can reduce soil N losses via altering cation exchange capacity, adsorption by surface functional groups^44,66^, by physical entrapment during field aging^67^, by reducing leaching and volatilization^68^. Based on these properties several recent studies have suggested nitrogen enrichment of biochar^69–71^ to utilize its biochar retention/sorption/capture property as a slow N release strategy^72^. Soil mineral N (NO_3_^−^ and NH_4_^+^) monitored at the harvest of first (cotton) and second crop (wheat) season in the present study at different soil depths showed greater N retention in the top-soil compared to sub-soil at 30–60 cm depth. The results are in agreement with Haider et al.^52^ where they found reduced nitrate leaching in a temperate sandy soil under field conditions. There was no visible residual effect on soil mineral N leaching in the following season in our study. It is attributed to reduced capacity of biochar to further sorb/capture mineral N as indicated by Beusch et al.^73^ where they found the biochar nitrate leaching reduction capacity was declined to half in the second period of their investigation. Thus, in this case our hypothesis of slow N release due to N enrichment of biochar was correct at least for the first cropping season, when AM and NeB were applied in combination compared to NeB application only. Such a reduction in N leaching or increased retention due to biochar addition with organic manures can also reduce nitrous oxide (N_2_O) emissions, which is a powerful greenhouse gas^74,75^. Reduced nitrate leaching due to biochar addition has potential to increase cotton productivity if biochar is successively applied in longer term^76^. Our results together with other findings in dry arid region of Pakistan^77^ towards biochar based slow N release carbon fertilizers.

### Influence of acidified manure and nitrogen-enriched biochar application on phosphorous availability in arid soil

The P mobility in different soil types around the globe is a matter of critical importance and complexity. Organic substances are recommended to prevent P sorption in soils and to enhance P recyclability^78^. Therefore, different organic amendments like acidified biochar^49^, or organic manures^78^ are recommended to improve soil P availability. The Olson’s P was also increased (54.6%) in the present study by the application of AM and NeB compared to control supplied with inorganic P from diammonium phosphate (DAP) fertilizer. The increase in Olson’s P availability was followed by AM (35.7%) and then by the NeB (18.9%). The results of increased Olson’s P due to AM or its combination application with NeB in the present study are in agreement with^79^ and^80^ where they found greater P availability in an alkaline soil due to reduced soil pH by the application of manure. However, there was no residual effect of organic amendments on Olson’s P in the second season. In principle, the organic amendments serve as blocking agents on P sorption sites due to their organic acids.

### Soil carbon dioxide emission as influenced by the application of acidified manure and nitrogen-enriched biochar

Application of biochar along with organics ‘compost’ increases soil microbial activity^81,82^ leading to greater greenhouse gases (GHGs) emission^83^. Acidification of manure (mainly studied are liquid manures) down to pH 6–6.5 has shown potential to reduce GHGs emissions^84^. The acidification with H_2_SO_4_ to pH 5.2–5.5 decreased methane emission by 68% and ammonia by 62%^85^. However, in the present study, soil CO_2_ emission was increased by the application of AM (in solid form) which may be attributed to greater availability of labile carbon, higher decomposition, and increased microbial respiration rate during the first 18 days of the study. The emission rate started declining from day 19 to 36 days of incubation. However, the net cumulative CO_2_ emission was reduced when AM was applied in combination with NeB due to stabilizing effect of biochar on manure^86^, but remained higher than the control soil without any manure or biochar amendment. A similar effect of compost and biochar, whether applied alone or in combination on soil CO_2_ emission was reported by other studies as well. Hence, our results suggest that the net impact of greater labile carbon availability from manure can be stabilized by its co-application with nitrogen-enriched biochar. Furthermore, these advances in waste management may lead to increased carbon sequestration as suggested by Schulz et al.^42^ under greenhouse experimental conditions where the combined application of biochar and manure increased soil carbon retention.

## Conclusion

We found significant yield improvements only in the first season (cotton) by the application of acidified manure (AM) alone or in combination with N enriched biochar (NeB). The clear yield improvements due to the combined application of AM and NeB in the first crop were associated with increased plant N, P, and K uptake. Furthermore, the co-application of AM and NeB increased mineral N (NO_3_^−^ and NH_4_^+^) in the topsoil during the first cropping season. However, the effects lasted only for the first growing season and did not extend to the second crop (wheat), despite a greater N and P uptake by the application of NeB. In this study pre-N enrichment and acidified manure corrected potentially the N sorption by biochar and synergized the effects at least for the first growing season under alkaline soil conditions. The lack of a persistent positive effect besides the initial effect of a one-time application of AM and NeB suggests that soil properties play a major role in the long run as a reduction in soil pH in microsites might have helped in the first crop, but the positive effect vanished within a year. The initial increase in soil CO_2_ emission by the application of AM, while the overall reduction in cumulative CO_2_ emission by the co-application of AM-NeB indicates the stabilizing effect on soil C/N ratios and microbial activity. To sum up, co-application of acidified manure and N enriched biochar can be a strategy to achieve short-term agronomic benefits.

## Data Availability

All data generated or analysed during this study are included in this published article.
